# Lightweight Driver Monitoring System Based on Multi-Task Mobilenets

**DOI:** 10.3390/s19143200

**Published:** 2019-07-20

**Authors:** Whui Kim, Woo-Sung Jung, Hyun Kyun Choi

**Affiliations:** Electronics and Telecommunications Research Institute, 218 Gajeong-ro, Yuseong-gu, Daejeon 34129, Korea

**Keywords:** lightweight, driver assistance, drowsiness, fatigue, distraction, PERCLOS, ECT, ECD, single-board computer, SBC, Raspberry pi

## Abstract

Research on driver status recognition has been actively conducted to reduce fatal crashes caused by the driver’s distraction and drowsiness. As in many other research areas, deep-learning-based algorithms are showing excellent performance for driver status recognition. However, despite decades of research in the driver status recognition area, the visual image-based driver monitoring system has not been widely used in the automobile industry. This is because the system requires high-performance processors, as well as has a hierarchical structure in which each procedure is affected by an inaccuracy from the previous procedure. To avoid using a hierarchical structure, we propose a method using Mobilenets without the functions of face detection and tracking and show this method is enabled to recognize facial behaviors that indicate the driver’s distraction. However, frames per second processed by Mobilenets with a Raspberry pi, one of the single-board computers, is not enough to recognize the driver status. To alleviate this problem, we propose a lightweight driver monitoring system using a resource sharing device in a vehicle (e.g., a driver’s mobile phone). The proposed system is based on Multi-Task Mobilenets (MT-Mobilenets), which consists of the Mobilenets’ base and multi-task classifier. The three Softmax regressions of the multi-task classifier help one Mobilenets base recognize facial behaviors related to the driver status, such as distraction, fatigue, and drowsiness. The proposed system based on MT-Mobilenets improved the accuracy of the driver status recognition with Raspberry Pi by using one additional device.

## 1. Introduction

Distracted and drowsy driving leads to thousands of deaths and injuries each year [1,2]. Over the decades, multiple research studies have been done based on artificial intelligence to find ways to reduce fatal crashes caused by the driver’s distraction and drowsiness. Despite the effort, driver monitoring systems have not become widespread because many studies are based on referring to the non-direct characteristics (e.g., steering wheel movement, the standard deviation of lane position), wearing intrusive equipment, or using high-performance processors. These research studies are categorized by the vehicle-based, behavioral, and physiological measurements [3].

Many automakers and researchers have adopted the vehicle-based measurement and have implemented the driver monitoring system using vehicle-based measurement because it delivers real-time performance, non-intrusive operation, and adaptability [4,5]. However, the vehicle-based measurement is not a solution specific to distraction and drowsiness because a variation of in-vehicle data can be the consequence of impaired driving, independent driving styles, road geometry, etc. [3,6]. The experiment of [7] showed a case where the driving pattern did not change relevantly, although the driver was drowsy. Furthermore, since vehicle-based measurement typically recognizes drowsiness after the drowsiness already reaches the last stage, warnings to prevent an accident could be generated too late [8].

The driver’s physiologic signals during driving are characteristic features that are closely related to recognizing distraction and drowsiness. The driver monitoring system using physiological measurement provides high accuracy and can be implemented in real time [9]. Despite those merits, commercializing the system is difficult because the equipment to collect the data of physiologic signals is usually huge, expensive, and intrusive [10]. There are several wearable devices, such as bands, watches, and headbands, to get physiologic signals, but they are not reliable sources to recognize the driver’s distraction and drowsiness. For these reasons, it is difficult to commercialize the driver monitoring system using physiological measurements.

Driver monitoring systems using behavioral measurement have been proposed as a compromise and have been actively researched for decades. Most of the research studies on the driver monitoring system using behavioral measurement have used hand-crafted features and classifiers of machine learning. However, the hand-crafted features are usually low-level features and unsatisfactory to handle object appearance variations, such as pose, illumination, and orientation [11,12]. It is too difficult to select the appropriate features and classifiers according to the dataset’s characteristics [11]. After the celebrated victory of AlexNet at the ILSVRC2012 (IMAGENET Large Scale Visual Recognition Challenge 2012) [13], deep-learning with the network for learning features has been actively researched and has achieved an excellent classification performance in various fields. As shown in Table 1, many recent studies of the driver monitoring system using behavioral measurement have used deep-learning algorithms.

However, since deep-learning algorithms require much memory and computation, most studies related to the deep-learning-based driver monitoring system utilize a high-performance CPU or GPU. Furthermore, most studies have a hierarchical structure that performs face detection, eye or mouth detection, and driver status (distraction or drowsiness) recognition in order. Not only does a hierarchical structure require higher specifications of CPU or GPU, but also the driver status may not be properly recognized if the driver’s face is not detected or is detected incorrectly. Although tracking algorithms have been used to address this problem, as of yet, there is no absolute solution for accurately detecting and tracking the driver’s face. Face detection and tracking can affect the system in terms of speed and accuracy.

In [14], Park et al. proposed a method that does not have a hierarchical structure and showed that the driver’s drowsiness could be directly recognized from the primary image without significantly decreasing the performance. Although AlexNet and VGG [15] models used in [14] have relatively shallow layers, their many parameters require much memory. In the experiment of [16], AlexNet showed the performance of 4.5 Frames Per Second (FPS) when five Raspberry Pis were used and 0.5 with VGG when nine Raspberry Pis were used. This shows that the driver monitoring systems based on deep-learning models such as AlexNet, VGG, and FlowNet [17] used in [14] had difficultly achieving real-time performance.

To solve this problem, we propose a Multi-Task Mobilenets (MT-Mobilenets)-based lightweight driver monitoring system that uses a resource sharing device such as the driver’s mobile phone. Our previous work [18] showed it is possible to recognize driver’s facial behaviors related to driver status recognition without relying on face detection and tracking by using Mobilenets, which is smaller and faster than AlexNet, VGG, and FlowNet. MT-Mobilenets is an improved method from the Mobilenets of the previous research. MT-Mobilenets uses a single Mobilenets without face detection and tracking to recognize facial behaviors independently, such as head direction, eye closure, and mouth opening. With facial behaviors recognized in the image, we defined the driver status, such as distraction and drowsiness. Thus, it is possible to recognize both distraction and drowsiness based on one MT-Mobilenets, unlike most other studies that focused on recognizing either distraction or drowsiness. With Raspberry Pi, a Single-Board Computer (SBC), the FPS of MT-Mobilenets is still not accurate enough to recognize the driver status. To alleviate this problem, we propose the lightweight driver monitoring system using a resource sharing device in a vehicle. The resource sharing device connected to the proposed system shares computing resources. The proposed system can obtain additional recognition results based on these shared resources. The multi-task MobileNets-based lightweight driver monitoring system increases FPS and improves accuracy by using a resource sharing device in the vehicle, such as the mobile devices of the driver or passengers.

The content of this article is organized into the following sections. Section 2 describes the dataset. Section 3 describes the proposed lightweight driver monitoring system. Section 4 contains the analysis and explanations of our experiments. Finally, Section 5 presents the conclusion of our research and the introduction of future work.

## 2. Dataset

### 2.1. Camera

**Figure 1:**
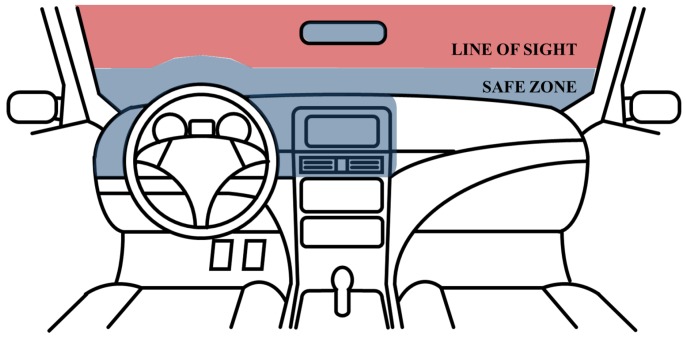
“Safe zone” and “line of sight” in the Anti-Distracted Driving Act.

In the Anti-Distracted Driving Law, enforced by The Department of Transportation in the Philippines, the area where the driver’s sight should not be disturbed is defined as the “line of sight”, and other areas where it is prohibited to install in-vehicle infotainment devices are defined as “safe zone” [28]. As shown in Figure 1, the areas, which are the top of the center-fascia, the dashboard near the driver, and the area up to four inches from the dashboard, defined as the “safe zone”, are not obstructing the driver’s “line of sight” when an in-vehicle infotainment device is mounted. For safety, placements of mobile phones and other electronic devices are allowed in the “safe zone”.

**Figure 2:**
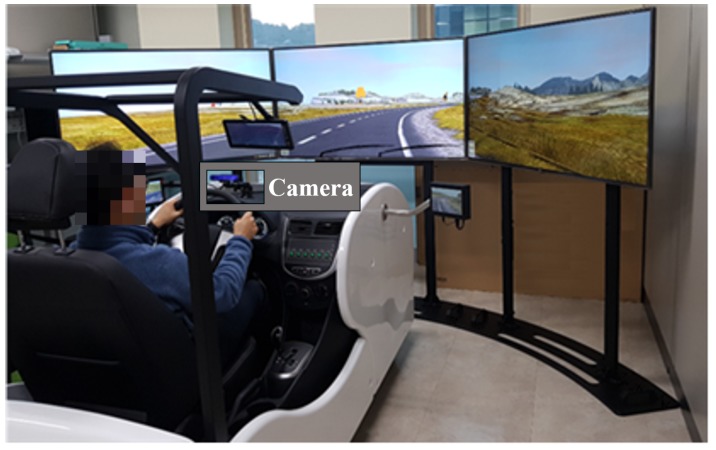
Driving simulator.

Many research studies have taken the installation of experimental equipment in the “safe zone” into consideration to ensure safe driving. In many driver monitoring research studies, a monitoring camera was installed near the rear-view mirror, near the sun visor, on the A-pillar, or on the dashboard to avoid obstructing the driver’s sight. A camera, placed near the rear-view mirror or the A-pillar, has the advantage that it is easy to install without occlusions caused by steering wheel manipulation, but only limited facial information, such as only one side of the driver’s profile view, is acquired. On the other hand, when installing a camera on the dashboard, it is necessary to consider the driver’s sight and occlusions caused by steering wheel manipulation. Although occlusions may occur, there is an advantage that facial information can be sufficiently obtained by capturing the image from the front of the driver. To take advantage of these features, as shown in Figure 2, we installed a camera on the dashboard that did not disturb the view of the driver.

### 2.2. Definition of the Driver Status

**Figure 3:**
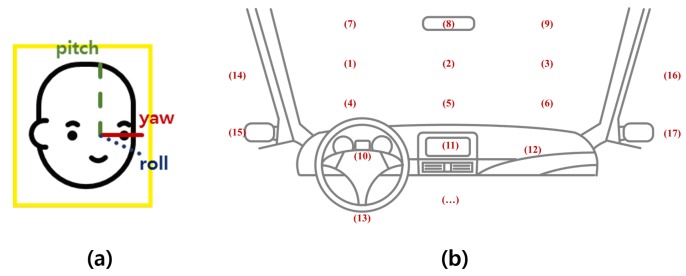
Head direction estimation (**a**) and gaze zone detection (**b**) in the related works.

**Figure 4:**
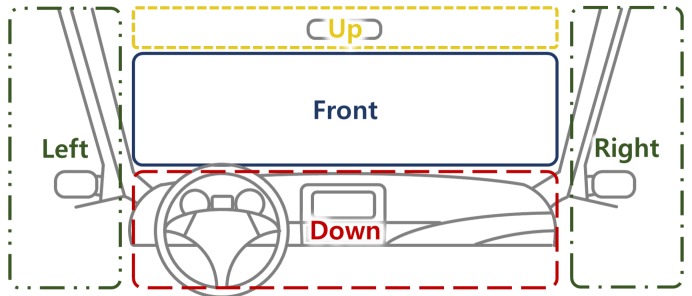
Head direction estimation in this paper.

A driver’s distraction is mainly recognized based on estimating head direction (yaw, pitch, and roll) and the gaze zone. Previous studies have focused on accurately estimating the type of driver’s gaze in subdivided gaze zones, as shown in Figure 3, or reducing the quantifying error in the estimation of the driver’s head direction including yaw, pitch, and roll for distraction detection [19,20]. However, these approaches require many computational resources for precisely measuring the head direction values of yaw, pitch, and roll and the gaze zone of the driver, which is too detailed for only distraction detection. In this paper, to obtain valid measurement results while consuming light computing resources, we separate the driver’s direction into five regions as shown in Figure 4 and define distraction as when the driver’s direction is maintained in a non-front zone for more than a certain time period.

In research studies on the driver monitoring system, drowsiness (or fatigue) is categorized into 2, 5, or 9 levels. Analyzing changes in driver status is difficult when drowsiness is categorized into two levels. In [29], the driver’s fatigue was specifically categorized into nine levels, as shown in Table 2. Changes in driver status can be analyzed. However, the definition of nuance among each level of fatigue is ambiguously described as +/0/−. Furthermore, indicators including yawning and head-nodding are unclear. There are too many indicators to use in light computing systems. Therefore, we categorized fatigue and drowsiness into four levels by eliminating or redefining not clearly-defined indicators and nuances, as shown in Table 2.

We determined the status of the driver using the factors including PERCLOS, duration of eye closure, mouth opening, and head direction. The duration ($D_{i,x}$) is defined as the cumulative time when $y_{i,x}$ is consecutively true, as shown in Equation (1). Because the processing time is inconsistent due to communication delay and packet loss, PERCLOS is defined as the proportion of the eyes closed time within a predefined time (*T*) as shown in Equation (2). $y_{i,x}$ means $i^{\mathrm{th}}$ frame’s true or false value of eye closure, mouth opening, and one-hot encoded head directions within total frames (*L*). $\tau_{i}$ means the processing time of the $i^{\mathrm{th}}$ frame.

(1) $$\begin{array}{c} D_{i,x}=\left\{\begin{array}{c} D_{i-1,x}+\tau_{i},\;\;\;\;\;if\;y_{i,x}\;is\;true\;\;\;\\ 0,\;\;\;\;\;\;\;\;\;\;\;\;\;\;\;\;\;\;if\;y_{i,x}\;is\;false \end{array}\right.\\ \left(i\in \{1,2,3,\ldots ,L\},\;x\in \{eye\;closure,\;mouth\;opening,\;head\;directions\}\right) \end{array}$$

(2) $$\begin{array}{c} PERCLOS=\frac{1}{N}\sum_{i=n}^{m}y_{x,i}\times \tau_{i},\;\;\;\;\;if\;\sum_{j=n}^{m}\tau_{j}\leq T\\ \left(x\in \{eye\;close\}\right) \end{array}$$

**Figure 5:**
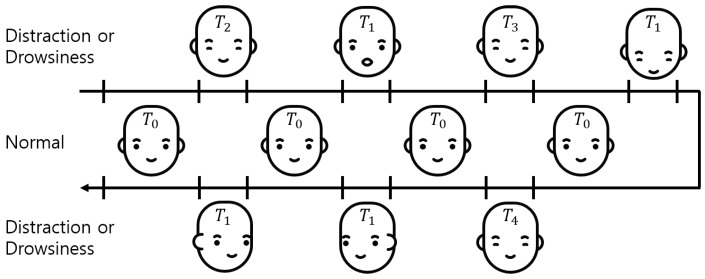
Acquisition procedure of the time-series data.

We acquired the time-series data for the evaluation dataset, as shown in Figure 5. The driver status was determined by using the time-series data, Equation (1), and Equation (2). After one status is determined, each status can be occluded when another status is determined within a predefined time (*T*). For an independent evaluation of each distraction, fatigue, and drowsiness status, the awake status was inserted between each status for longer than the predefined time ($T_{0}$). For each subject, the time series data were obtained in the order of light and medium fatigue, drowsiness (strong fatigue), and distraction. The acquisition time of each status was defined, as shown in Equation (3), in accordance with Table 2.

(3) $$X_{i}=\left\{\begin{array}{c} T_{0}\in \;\;\;\;\;\;\;\;\;\;\;\;\left[T,\infty \right)\\ T_{1}\in \;\;\;\;\;\left[2.0\;s,\infty \right)\\ T_{2}\in \left[0.5\;s,1.0\;s\right)\\ T_{3}\in \left[1.0\;s,1.5\;s\right)\\ T_{4}\in \left[1.5\;s,2.0\;s\right) \end{array}\right.$$

### 2.3. Generation of the Ground Truth of Facial Direction and Expression

The rough ground truths of facial behavior, required to determine the ground truths of driver status, were gathered by OpenFace 2.0 [30,31,32,33]. The procedure is briefly described below. The driver’s face is detected by the Multi-Task Convolutional Neural Network (MTCNN) face detector, which was trained with WIDER FACE and CelebA. In contrast to other facial detectors, which use HoG-SVM, Haar–Cascade, etc., MTCNN can detect frontal, profile, and highly-occluded faces by using a single detector. The bounding box of the detected face is used to detect facial landmarks and facial behavior recognition.

**Figure 6:**
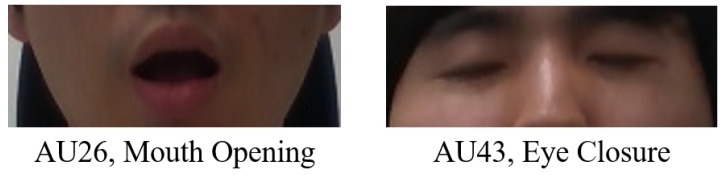
Action Unit 26 (AU26) and AU43 of the facial action coding system [34,35].

**Figure 7:**
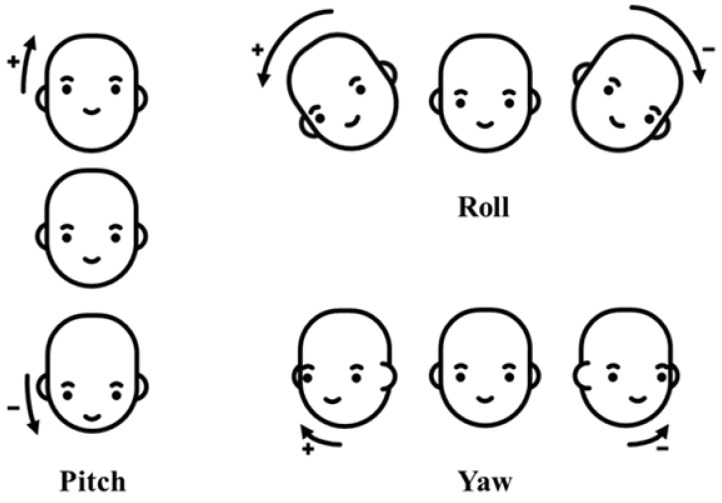
Yaw, roll, and pitch.

The Convolutional Experts Constrained Local Models (CE-CLM) detect 2D Cartesian coordinates of 68 facial landmarks. The CE-CLM internally has data on the 3D coordinates of facial landmarks, and head direction (yaw, pitch, and roll) is estimated by the perspective-n-point algorithm. Using facial landmarks detected by CE-CLM and HoG features extracted from the aligned detected face image of 112×112, the intensity and presence of facial Action Units (AU#) are estimated by support vector regression. Among the outputs of OpenFace 2.0, the data of yaw, pitch, roll, AU26, and AU43 were used, as shown in Figure 6 and Figure 7.

The camera was aligned upward at about 10 degrees to capture the driver’s face at the center of the image because the camera was mounted on the dashboard and located below the driver’s face, as shown in Figure 2. The head angles measured experimentally in the driving simulator for the 12 subjects were ±15, 25, and −5 degrees for the side view mirrors, sun visor, and the bottom of the cluster, respectively. In consideration of these angles, the head directions of the driver were labeled using Equation (4) with the estimated yaw, pitch, and roll. *P*, θ, and ϑ in Equation (4) mean head directions, angles, and camera alignment angle.

With OpenFace 2.0, the intensity of facial expressions was normalized in the scores between zero and five (real number). For the subjects, the intensity of AU26 and AU43 corresponding to mouth opening and eye closure was estimated to be higher than a value between two and three. Mouth opening and eye closure were labeled with slightly different thresholds depending on the subjects.

(4) $$Head\;Direction=\left\{\begin{array}{c} P_{front},\;\;\left|\begin{array}{c} \theta_{pitch}-\vartheta_{pitch} \end{array}\right|\leq \theta_{\tau }\;AND\;\left|\begin{array}{c} \theta_{yaw} \end{array}\right|\leq \theta_{\tau }\\ P_{up},\;\;\;\;\;\;\;\;\;\;\;\;\;\;\;\;\;\;\;\;\;\;\;\;\;\;\;\;\;\;\;\;\;\;\;\;\;\;\;\;\;\;\;\;\;\theta_{pitch}>\;\;\;\theta_{\tau }\\ P_{down},\;\;\;\;\;\;\;\;\;\;\;\;\;\;\;\;\;\;\;\;\;\;\;\;\;\;\;\;\;\;\;\;\;\;\;\;\;\;\;\;\;\theta_{pitch}<-\;\theta_{\tau }\\ P_{right},\;\;\;\;\;\;\;\;\;\;\;\;\;\;\;\;\;\;\;\;\;\;\;\;\;\;\;\;\;\;\;\;\;\;\;\;\;\;\;\;\;\;\;\theta_{yaw}>\;\;\;\theta_{\tau }\\ P_{left},\;\;\;\;\;\;\;\;\;\;\;\;\;\;\;\;\;\;\;\;\;\;\;\;\;\;\;\;\;\;\;\;\;\;\;\;\;\;\;\;\;\;\;\;\theta_{yaw}<-\;\theta_{\tau } \end{array}\right.$$

**Figure 8:**
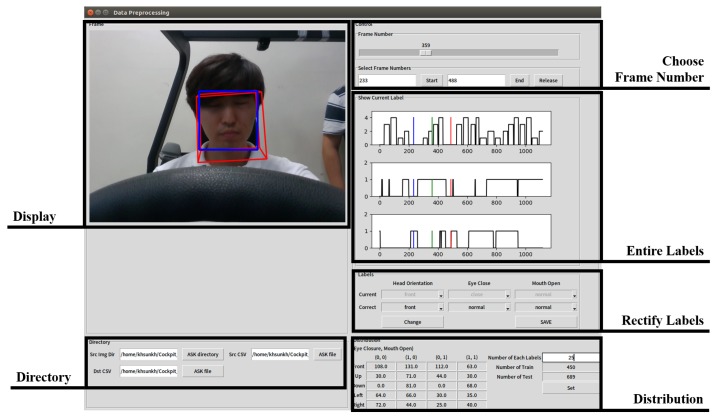
Correcting labels program.

In many cases, labels were incorrectly assigned due to errors in the process of detecting the face or localizing landmark points. To fix this, we built a program for us to correct the error in the wrongly-assigned labels using the Tkinter Python GUI library, as shown in Figure 8. “Display” shows the selected frame image with the result obtained from OpenFace. Wrong labels assigned to the selected frames can be rectified on “rectify labels”, and the results after modification are displayed on “entire labels”. The learning and test data can be randomly selected from the whole data by specifying the number of each label in the “distribution”.

## 3. Lightweight Driver Monitoring System Configuration

**Figure 9:**
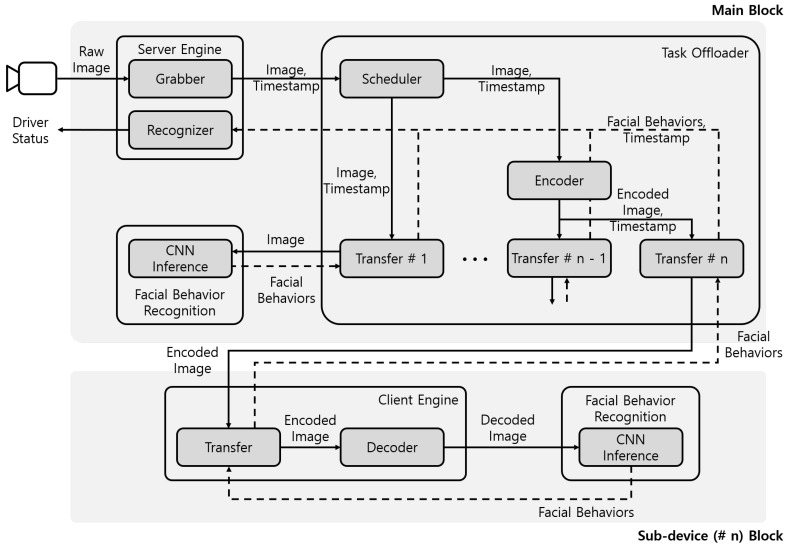
Configuration of the proposed system.

The proposed system consisted of the main block implemented on the in-vehicle SBC, and sub-device blocks implemented on the in-vehicle resource sharing devices such as the driver’s mobile device, as shown in Figure 9. The main block included a server engine module, a task offloader module, and a facial behavior recognition module. The server engine module performed the functions of image acquisition, image transmission, and driver status recognition. The task offloader module managed the schedule among facial behavior recognition modules. The task offloader module also delivered images from the server engine module to the facial behavior recognition module in the main block, and it delivered encoded images to sub-device blocks. The sub-device blocks included a client engine module and the facial behavior recognition module. The client engine module decoded received images from the main block and delivered the decoded image to the facial behavior recognition module in the sub-device block. The facial behavior recognition module recognized the facial behaviors, such as head directions, eye closure, and mouth opening. The facial behavior recognition module of sub-device blocks performed identical functions as that of the main block. The sub-device blocks recognized the facial behaviors of some images, which were not processed by the main block, and transferred the facial behaviors recognition results to the main block. The main block accumulated facial behavior recognition results from the main block and the sub-device blocks and recognized the status of the driver on the in-vehicle SBC.

Since Wi-Fi uses the ISM band, communication performance may be affected depending on the surrounding environment. This problem can be classified into interference caused by a signal received from outside the vehicle and interference generated inside the vehicle. However, the interference from the outside of the vehicle does not have a great influence on the communication because it gives a relatively small interference to the inside of the vehicle made of an iron and steel structure. In order to minimize the influence of the interference caused by the competition in the vehicle, we used high-priority IEEE 802.11e QoS (Quality of Service). Nevertheless, in order to minimize the delay caused by the high traffic state, the scheduler was designed considering the transmission delay time by selecting the image to be transmitted through the communication state prediction model [36] when receiving the request from the sub-device block.

### 3.1. Server Engine Module

**Figure 10:**
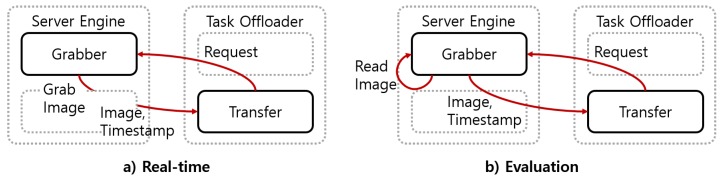
Operation of the server engine module in the real-time and evaluation experiment.

The server engine module performed the functions of image acquisition, image transmission, and driver status recognition. The server engine consisted of grabber and recognizer components. When the grabber component received a request from the task offloader module, it transmitted the image acquired from the camera and the timestamp of the image. To minimize transmission delays via Wi-Fi, the grabber component preprocessed the images through center cropping, resizing, and normalizing. The recognizer component accumulated facial behavior recognition results and recognized driver status from the accumulated results. In the real-time implementation, the server engine module worked whenever it received the request and the behavior recognition results, as shown in Figure 10a.

For the evaluation, we needed to use datasets that had images and the ground truth. The server engine module needed to transmit the image to be processed next and recognized the driver status considering the processing time of the subsequent modules. In the experiment, the server engine module worked, as shown in Figure 10b. The grabber component loaded the saved image sequentially, regardless of the request from the task offloader module. It transmitted the loaded image whenever it received a request from the task offloader module. It loaded each image at a time interval calculated by Equation (5). The facial behaviors that were not recognized in unprocessed images were assigned with the last results received from the task offloader module. With the time-series data of the facial behaviors’ recognition results, the recognizer component recognized the driver status.

In Equation (5), $D_{load}$ means the delay to load the next image. $t_{n}$ means the timestamp of the $n^{\mathrm{th}}$ image. $T_{load}$ means the time to load the current image.

(5) $$D_{load}=t_{n}-t_{n-1}-T_{load}$$

### 3.2. Task Offloader Module

**Figure 11:**
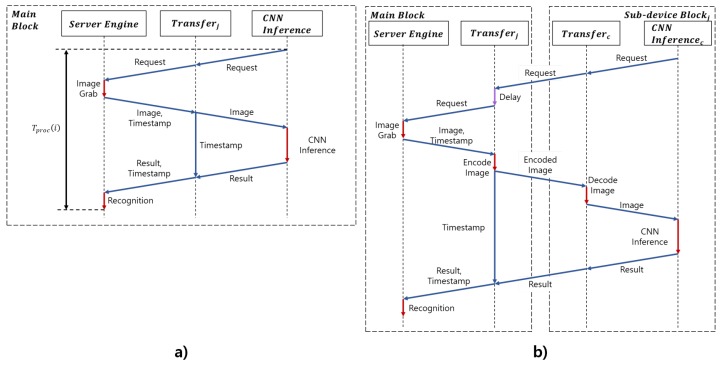
(**a**) Procedure in the main block. (**b**) Procedure between the main and sub-device blocks.

The task offloader had transfer and encoder components to deliver images. The task offloader had one transfer component by default. When a resource sharing device in a vehicle created a sub-device block, it connected the main block via Wi-Fi. The task offloader module of the main block assigned an ID to the device and created a new corresponding transfer component. Each transfer component received the pre-processed image from the server engine module, along with a timestamp corresponding to the image. The first transfer component module transmitted the image to the facial behavior recognition module of the main block and received recognition results from the facial behavior recognition module. For another transfer component, the encoder component encoded the image in the byte format, and the transfer component delivered the encoded image over the sockets to a sub-device block via Wi-Fi. Whenever transfer components received the facial behavior recognition results, they delivered both the recognition results and the timestamp to the server engine module. Figure 11 shows the procedure of the proposed system.

The task offloader module managed the schedule among transfer components by a scheduler component. The task offloader consisted of a scheduler component, an encoder component, and transfer components. The scheduler component of the task offloader scheduled facial behavior recognition modules of the main block and the sub-device blocks to not process adjacent images in the following step: First, we calculated the average time ${\bar{T}}_{proc}$ to recognize the driver status from the $N^{\mathrm{th}}$ image to the current $M^{\mathrm{th}}$ image in the main block. Then, the average time was divided by the number of transfers (*L*). The delay (Delay) as calculated by multiplying the $\frac{1}{L}{\bar{T}}_{proc}$ and the transfer’s number *j* as shown in Equation (6). $T_{proc}\left(i\right)$ represents the processing time from making a request to performing recognition, as shown in Figure 11a. Finally, the task offloader module requested the next image after ${Delay}_{j}$ and delivered the image though the $j^{\mathrm{th}}$ transfer.

(6) $${Delay}_{j}=\frac{j}{L}{\bar{T}}_{proc},\;\;\;\;\;\;\;{\bar{T}}_{proc}=\frac{1}{M-N}\sum_{i=N}^{M}T_{proc}\left(i\right)$$

### 3.3. Client Engine Module

The client engine module had transfer and decoder components. The transfer component delivered encoded images from the main block to the decoder component. The decoder component decoded the received encoded image and delivered the decoded image to the facial behavior recognition module of the sub-device block. The transfer component received the recognition results from the facial behavior recognition module. It delivered the recognition results to the main block.

### 3.4. Facial Behavior Recognition Module

The facial behavior recognition modules recognized the facial behavior from the driver image, such as head direction, eye closure, and mouth opening. These modules of the main block and the sub-device blocks used the same identical functions. A CNN inference component of the facial behavior recognition module used a previously-trained CNN model. This component performed only the model inference after first loading the pre-trained CNN inference model. The facial behavior recognition modules transferred the recognition results of the CNN inference component to each corresponding transfer component.

**Figure 12:**
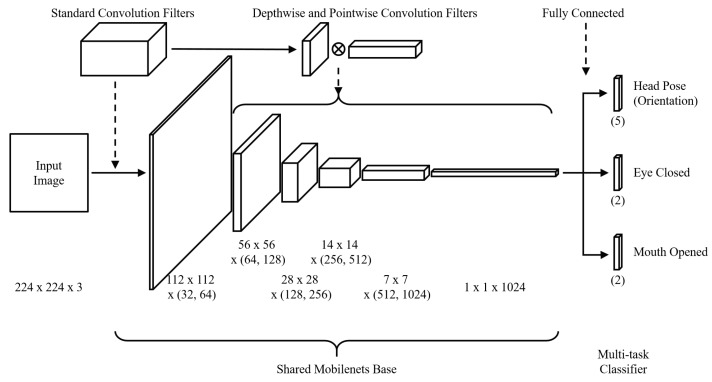
Multi-task Mobilenets.

We used MT-Mobilenets to recognize facial behaviors, such as head direction, eye closure, and mouth opening. Figure 12 describes the structure of MT-Mobilenets. MT-Mobilenets enables recognizing the facial behaviors from the image without the functions of face detection and tracking. Using MT-Mobilenets has the following advantages over traditional driver monitoring methods: First, MT-Mobilenets is driven by Mobilenets, which shows a relatively low computational latency and high accuracy in the mobile environment. Besides, MT-Mobilenets does not depend on whether the driver’s face, eye, and mouth are precisely detected. Finally, facial behaviors, including head direction, eye closure, and mouth opening, can be simultaneously recognized from one shared Mobilenets base.

## 4. Experiment

### 4.1. Experimental Environment and Data

A desktop computer with four NVIDIA GTX Titan Xp was used for training, and Raspberry Pi was used for testing. Ubuntu 16.04 and Raspbian 9.1 for OS and Tensorflow for the deep-learning framework were used. Raspberry Pi has a four-core Cortex A53 CPU and does not have a GPU. Raspberry Pi has 5.47 GFLOPS performance, which was calculated by the High-Performance Computing Linpack Benchmark in double precision [37].

This study used a driving simulator to obtain the data for the experiment. This simulator was built by using the driver’s seat of an actual vehicle (Hyundai Accent). The camera could be installed and tested in consideration of the actual structure of the vehicle interior. The camera used in the experiment was mounted on the dashboard in front of the driver to obtain sufficient facial data. The CREATIVE BlasterX Senz3D camera produces images with a resolution of 640×480 pixels, which were retrieved through the USB 3.0 interface at a frequency of 30 Hz. Furthermore, the retrieval time of each image was recorded in the UTC.

Twelve subjects ranging in age from 20–50 participated in this experiment. A total of 38,945 images were obtained from the twelve subjects, of which 20,000 images from six subjects were used for training MT-Mobilenets of the facial behavior recognition module, and 18,954 images from the others were used for testing the entire system. As mentioned in Section 2.3. The dataset for training MT-Mobilenets consisted of three facial behavior recognition tasks: head direction, eye closure, and mouth opening. Each task of facial behavior recognition had two or five classes, as shown in Table 3. There were 20 cases. Because balancing the number of samples between each class is generally recommended for training a model with supervised learning, each case was composed of 1000 images obtained from eight subjects. For the test, the subjects were asked to show facial behavior corresponding to distraction, fatigue, and drowsiness in the order shown in Figure 5. The test dataset had 18,945 images, including 8254 images labeled with “distraction”, “fatigue”, and “drowsiness”.

### 4.2. Training of MT-Mobilenets and Testing of the Proposed System

There were three sets of ground truths ${\hat{y}}_{i}$ corresponding to three tasks of facial behavior recognition, which had different lengths $l_{n}$ in classes. For the results $y_{i}$ of facial behavior recognition by MT-Mobilenets, the cost $C_{n}$ of each task of the facial behavior recognition was calculated by the Softmax cross-entropy Equation (7), and the total cost $C_{total}$ was calculated as the sum of the costs of *N* facial behavior recognition tasks, as shown in Equation (8). The Adaptive Moment estimation (Adam) method was used for training MT-Mobilenets. For hyper-parameters of Adam, the learning rates $l_{r}$, $\beta_{1}$, $\beta_{2}$, and ε were set as 0.01, 0.9, 0.999, and $1e^{-8}$, respectively. The parameters (*m*, $\hat{m}$, *v*, and $\hat{v}$) of Adam were updated by Equation (9), and the weights *w* of MT-Mobilenets were updated by Equation (10).

(7) $$\begin{array}{c} C_{n}=-\frac{1}{l_{n}}\sum_{i=1}^{l_{n}}\left\{y_{i}\ln{\hat{y}}_{i}+\left(1-y_{i}\right)\ln\left(1-{\hat{y}}_{i}\right)\right\} \end{array}$$

(8) $$\begin{array}{c} C_{total}=\sum_{n=1}^{N}C_{n} \end{array}$$

(9) $$\begin{array}{cc} m_{w}\left(t\right)=\beta_{1}m_{w}\left(t-1\right)+\left(1-\beta_{1}\right)\nabla_{w}C\left(t\right) & v_{w}\left(t\right)=\beta_{2}v_{w}\left(t-1\right)+\left(1-\beta_{2}\right){\left(\nabla_{w}C\left(t\right)\right)}^{2}\\ {\hat{m}}_{w}=\frac{m_{w}\left(t\right)}{1-{\left(\beta_{1}\right)}^{t}} & {\hat{v}}_{w}=\frac{v_{w}\left(t\right)}{1-{\left(\beta_{2}\right)}^{t}} \end{array}$$

(10) $$\begin{array}{cc} w\left(t\right)=w\left(t-1\right)-l_{r}\left(t\right)\frac{{\hat{m}}_{w}}{\sqrt{{\hat{v}}_{w}}+\varepsilon } & l_{r}\left(t\right)=l_{r}\left(0\right)\frac{\sqrt{1-{\left(\beta_{2}\right)}^{t}}}{1-{\left(\beta_{1}\right)}^{t}} \end{array}$$

**Figure 13:**
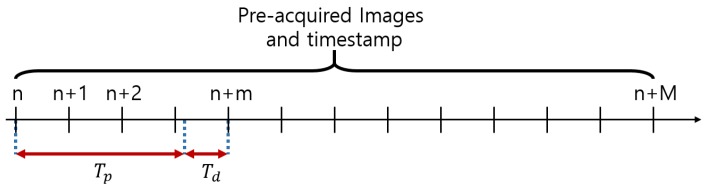
Sequence of input images.

As mentioned in Section 2.2, the processing time of the system could influence the result of driver status recognition. To analyze the accuracy of driver status recognition according to the processing time of each system, the next image to be processed needed to be selected by considering the processing time and fed into the facial behavior recognition module, as shown in Figure 13. After processing time $T_{p}$ for facial behavior and status recognition, the next image was input after the delay of $T_{d}$ as Equation (11). *n* is the number of images currently being processed, and *m* is the number of images that the grabber of the server engine module has read while the $n^{\mathrm{th}}$ image is being processed. T(n) means the timestamp of the $n^{\mathrm{th}}$ image.

(11) $$T_{d}=T\left(n+m\right)-T\left(n\right)-T_{p}$$

The data of each subject were evaluated to ensure the independence of the data. Then, the results for each subjects were combined, and the accuracy of all data was calculated. The accuracy of each task of the status recognition *c* is the ratio of $N_{c,true}$ to the number of total frames $N_{total}$, as shown in Equation (12). $N_{c,true}$ represents the number of the same values of the output $y_{c,n}$ and ground truth ${\hat{y}}_{c,n}$.

(12) $$\begin{array}{ccc} {Accuracy}_{c}=\frac{N_{c,true}}{N_{total}} & N_{c,true}=\sum_{n=1}^{N_{total}}O_{c,n} & O_{c,n}=\left\{\begin{array}{cc} 1 & \;\mathrm{if}\;y_{c,n}=={\hat{y}}_{c,n}\\ 0 & \;\mathrm{otherwise}\; \end{array}\right. \end{array}$$

### 4.3. Result of the Experiment

**Figure 14:**
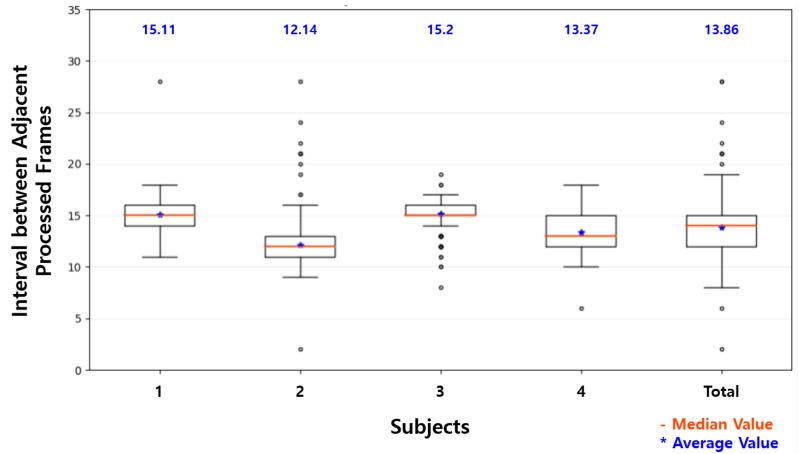
Interval distribution on the Raspberry Pi.

Table 4 shows a comparison of the processing time to recognize the driver status with Raspberry Pi and in the proposed system. The average FPS with Raspberry Pi was 2.15 FPS. The processing times of the inference in Raspberry Pi were irregular. This led to the irregular interval between the adjacent processed images, as shown in Figure 14, showing the box-plots of intervals between the adjacent processed images on a Raspberry Pi for four subjects. The average interval was 13.86 on Raspberry Pi.

**Figure 15:**
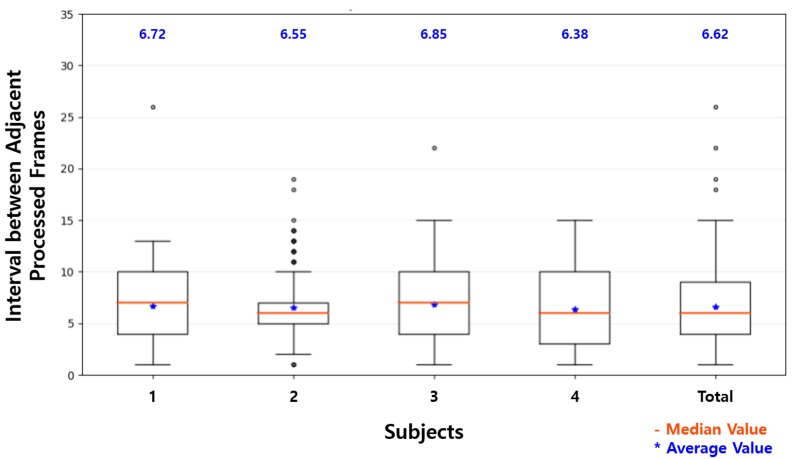
Interval distribution on the proposed system.

As shown in Figure 15, although there was some loss in the wireless communication, the average of frames per second was 4.47 FPS, which was more than twice that of a single Raspberry Pi by using the proposed system with one additional device. The average interval was decreased by more than half to 6.62. In the proposed system, the server engine module in the separate thread repeatedly captured images from the camera and determined the status of the driver whenever the results of the processed image were input. Because the facial behavior recognition modules in the main block and the sub-device block received the loaded image at the end of the process and sent the results of processing, these could analyze the next image without waiting for image acquisition and status recognition. This slightly improved the throughput of the system. The more additional devices would be used in the proposed system, the more effective the system would be. These minor improvements were due to the structure in which each module was processed in separate threads.

To evaluate the proposed system, a pre-trained model of MT-Mobilenets was used that recognized facial behaviors such as face direction, eye closure, and mouth opening. As shown in Table 5, the average accuracy of each task of the facial behavior recognition with Raspberry Pi was 94.74%, 72.49%, and 90.50%, respectively. While the driver was driving, the facial behaviors, such as changing face direction and mouth opening, occasionally continued for a relatively long time, and blinking of eyes frequently went on for a short time. The short blinking of eyes usually occurred in less than ten consecutive frames and was difficult to detect with the Raspberry Pi at an average of 13.86 intervals. The recognition accuracy of eye closure was much lower than that of face direction and mouth opening. With the proposed system showing decreased average intervals at 6.62, as shown in Figure 15, some of the short eye blinking that was not recognized with Raspberry Pi was recognized. This difference explains that the accuracy improvement of eye closure recognition was slightly larger than that of the recognition of face direction and mouth opening. The recognition accuracy of each task was 96.40%, 77.56%, and 93.93%, respectively, which were only slightly improved compared to the Raspberry Pi.

As shown in Table 6, although the accuracy of facial behavior recognition was only slightly improved, the proposed system had a significant impact on the recognition of driver status including distraction, fatigue, and drowsiness. The average accuracy of the driver status recognition on a Raspberry Pi was 85.85%, 74.09%, and 82.34%, respectively. With the proposed system using one additional device, the average accuracy of the driver status recognition was 98.96%, 84.89%, and 94.44%, respectively. By using the proposed system, the accuracy of the driver status recognition was significantly improved compared to the accuracy of the driver status recognition with Raspberry Pi and higher than the recognition accuracy of 83% of Panasonic’s aftermarket product released in 2017 [38].

This improvement in the accuracy of the driver status recognition was achieved by the proposed system with the reduced average intervals between the processed images. As shown in Table 7, which lists the representative intervals in Figure 14 and Figure 15, three-quarters of the processed frames were processed at more than 13 intervals on Raspberry Pi. Assuming the time difference between adjacent intervals was about 33 ms, as mentioned in Section 4.1, most frames were processed at one or two times per second. As shown in Table 2, the driver status, such as distraction, fatigue, and drowsiness, was defined in units of 0.5 s in this study. That is, one or no frame was processed every 0.5 s (a unit time of the status definition) on Raspberry Pi. On the other hand, all frames except outliers were processed at 15 or less intervals on the proposed system. The processed frames were processed once every 0.5 s. For half of the processed frames, two or more frames were processed every 0.5 s, and the accuracy of the driver status recognition for the frames was higher than that of the driver status recognition for the others. This shows that two or more frames should be processed within the minimum unit time of the status definition to achieve reasonable recognition accuracy in the lightweight system.

## 5. Conclusions

In this paper, we used Multi-Task Mobilenets (MT-Mobilenets), which had a multi-task classifier. The three softmax regressions that compose the multi-task classifier can simultaneously recognize facial behaviors including face direction, eye closure, and mouth opening from the shared Mobilenets base. Since MT-Mobilenets was trained with images and facial behavior labels without using face detection and tracking, the facial behaviors could be recognized directly from the images. The driver status, such as distraction, fatigue, and drowsiness, was recognized by the duration of the facial behaviors and PERCLOS. MT-Mobilenets that did not utilize face detection and tracking recognized the driver status at an average 2.146 FPS on a single Raspberry Pi. However, the number of obtained facial behaviors per second on the single Raspberry Pi was insufficiently accurate to recognize the driver status. To solve this problem, we proposed a lightweight driver status recognition system on the SB based on MT-Mobilenets. For most processed frames, the single Raspberry Pi processed one or no frames per 0.5 s, which is the minimum unit time of the driver status definition. The proposed system using one additional device processed more than one frame per 0.5 s and processed more than two frames per 0.5 s for half of the processed frames. Our experiments showed that the proposed system provided higher performance than the single Raspberry Pi.

The experiments showed that the images needed to be processed more than twice within the minimum unit time of the driver status definition to achieve a reasonable accuracy of the driver status recognition in an SBC such as Raspberry Pi. This could be satisfied by using one additional device in the proposed system based on Raspberry Pi. The proposed system showed an average accuracy of 11.0 percentage points higher than that of the single Raspberry Pi and higher accuracy than Panasonic’s aftermarket product released in 2017.

We expect that if the higher accuracy of the pre-trained model of facial behavior recognition is utilized, the proposed system will support a higher accuracy of driver status recognition. We plan to improve the recognition accuracy of the driver status recognition in the future by training MT-Mobilenets with a vast driver dataset. While this experiment used Raspberry Pi for a resource sharing device in a vehicle, we plan to verify the proposed system with a real device in the vehicle, such as the driver’s mobile phone.

## Figures and Tables

**Table 1:** Related works on the driver monitoring system.

		Fatigue or	Detection			Used	Environments		Maximum
Paper	Distraction	Drowsiness	Face	Eye	Landmark	Algorithms	CPU	GPU	FPS *
[19]	✓		✓		✓	VGG	H**	H	89.2
[20]	✓		✓			MTDNN	H	H	33.0
[21]		✓	✓		✓	MTCNN, AlexNet	Jetson TX1		14.9
[22]		✓	✓		✓	VGG	H	H	63.7
[23]		✓	✓		✓	MTCNN based on VGG	H	H	-
[24]		✓	✓		✓	Eye aspect ratio based on landmark	i.MX6Quad		16.0
[25]		✓	✓	✓		Spectral/linear regression	H	-	5.0
[26]		✓	✓		✓	Random forest classification	H	-	22.0
[27]	✓					AlexNet, VGG, GoogleNet, ResNet	Jetson TX1		14.0
[14]		✓				AlexNet, VGG, FlowNet	-	-	-

* FPS: Frames per second. ** H: High Performance.

**Table 2:** Definitions of the driver status.

Level	Facial and Behavioral Indicators in [29]	Level	Facial and Behavioral Indicators in Our Dataset
Awake	Fast eyelid closure, inconspicuous blink behavior, continuous switches of focus, upright sitting position, fast saccades, hand position on the steering wheel at the 10 and 2 o’clock	Awake	Otherwise
Light fatigue (−)	Prolonged eyelid closures of up to 0.5 s, tired facial expression,	Light fatigue	Mouth opening duration (>1 s) or eye closure duration (≥0.5)
Light fatigue (+)	yawning, rubbing/scratching of face, grimacing, tilted head		
Medium fatigue (−)	Prolonged eyelid closures (approximately 0.5–1 s),		
Medium fatigue (0)	eye staring/“glassy eyes” with long blinking pauses (>3 s),	Medium fatigue	Eye closure duration (≥1.5) or PERCLOS (≥0.1)
Medium fatigue (+)	stretching/lolling, eyes half closed		
Strong fatigue (−)	Very long eyelid closures (1–2 s), eye rolling, head-nodding		
Strong fatigue (+)		Drowsiness (Strong fatigue)	{Eye closure duration (≥1.5 s) and PERCLOS (≥0.2)} or
Very strong fatigue	Eyelid closures (>2 s),micro-sleep episodes, startling awake from sleep or micro-sleep		Look down duration (≥2 s)
Distraction	-	Distraction	Duration of looking in one direction other than the front (≥2 s)

**Table 3:** The tasks of the facial behavior recognition.

Facial Behavior	Normal	Abnormal			
**Head Direction**	Front	Up	Down	Left	Right
**Eye Closure**	Open	Close			
**Mouth Opening**	Close	Open			

**Table 4:** Comparison of the processing times.

		Processed Frames (Quantity)		Total	Frames Per Second (FPS)	
Subject	Total Frames	Raspberry Pi	Proposed System	Times (s)	Raspberry Pi	Proposed System
**1**	4668	308	692	156.77	1.97	4.41
**2**	4512	371	687	151.52	2.45	4.53
**3**	4916	322	716	165.19	1.95	4.33
**4**	4849	362	757	162.86	2.22	4.65
**Average**	4736	341	713	159.09	2.15	4.47

**Table 5:** Accuracy of the facial behavior recognition.

Facial Behavior	Face Direction	Eye Closure	Mouth Opening
**Quantity of Abnormal**	1903	2127	573
**Accuracy on Raspberry Pi**	94.74%	72.49%	90.50%
**Accuracy on Proposed System**	96.40%	77.56%	93.93%

**Table 6:** Accuracy of the driver status recognition.

	Distraction	Fatigue	Drowsiness
**Raspberry Pi**	85.85	74.09	82.34
**Proposed System**	98.96	84.89	94.44

**Table 7:** Representative interval.

	Raspberry Pi	Proposed System
**Maximum**	19	15
**Three Quarters**	15	9
**Two Quarters**	14	6
**One Quarter**	12	4
**Minimum**	8	1
